# JunD Regulates Pancreatic β-Cells Function by Altering Lipid Accumulation

**DOI:** 10.3389/fendo.2021.689845

**Published:** 2021-07-16

**Authors:** Kexin Wang, Yixin Cui, Peng Lin, Zhina Yao, Yu Sun

**Affiliations:** ^1^ Department of General Surgery, Qilu Hospital of Shandong University, Jinan, China; ^2^ Department of Endocrinology, Qilu Hospital of Shandong University, Shandong University, Jinan, China; ^3^ Institute of Endocrine and Metabolic Diseases of Shandong University, Jinan, China; ^4^ Center for Reproductive Medicine, Cheeloo College of Medicine, Shandong University, Jinan, China

**Keywords:** T2DM, pancreatic β-cells, lipotoxicity, JunD, lipid accumulation

## Abstract

The impairment of pancreatic β-cells function is partly caused by lipotoxicity, which aggravates the development of type 2 diabetes mellitus. Activator Protein 1 member JunD modulates apoptosis and oxidative stress. Recently, it has been found that JunD regulates lipid metabolism in hepatocytes and cardiomyocytes. Here, we studied the role of JunD in pancreatic β-cells. The lipotoxic effects of palmitic acid on INS-1 cells were measured, and JunD small-interfering RNA was used to assess the effect of JunD in regulating lipid metabolism and insulin secretion. The results showed that palmitic acid stimulation induced the overexpression of JunD, impaired glucose-stimulated insulin secretion, and increased intracellular lipid accumulation of β-cells. Moreover, the gene expression involved in lipid metabolism (Scd1, Fabp4, Fas, Cd36, Lpl, and Plin5) was upregulated, while gene expression involved in the pancreatic β-cells function (such as Pdx1, Nkx6.1, Glut2, and Irs-2) was decreased. Gene silencing of JunD reversed the lipotoxic effects induced by PA on β-cells. These results suggested that JunD regulated the function of pancreatic β-cells by altering lipid accumulation.

## Introduction

The prevalence of type 2 diabetes mellitus (T2DM) in the world is increasing yearly. Its complications, such as cardiovascular diseases, retinopathy, and nephropathy, have been imposed a heavy burden on public health (1–3). Insufficiency of insulin secretion of pancreatic β-cells and insulin resistance, due to prolonged lipotoxicity at least in part, are the basic characteristics of diabetes. Therefore, elucidating the potential mechanism of lipotoxicity on pancreatic β-cells dysfunction is crucial in the field of diabetic therapy.

Previous studies had demonstrated that T2DM could cause lipid accumulation in non-adipocytes, including hepatocytes and myocytes (4). Recent studies have indicated the existence of lipid and its associated proteins in human β cells (5, 6). As far as metabolic diseases are concerned, intracellular lipid accumulation is often caused by the imbalance of fatty acid synthesis, uptake, and hydrolysis, which eventually leads to the activation of cell apoptosis. Lipid deposition in pancreatic β-cells reduces insulin secretion (7), therefore, it is important to decipher the mechanism of intracellular lipid deposition in pancreatic β-cells.

Activator Protein 1 complex, which is composed of three Jun proteins (c-Jun, JunB, and JunD), four Fos proteins (c-Fos, FosB, Fra-1, and Fra-2), and four ATF proteins (ATF1-4, ATF-6, β-ATF, and ATFx) (8), plays a crucial role in regulating cell growth and metabolism. AP-1 member JunD modulates cell differentiation, proliferation, and apoptosis (9) and protects cells against oxidative stress by limiting the production of reactive oxygen species (10). JunD-/- mice exhibited a shortened life span and increased pancreatic angiogenesis (11). Besides, JunD regulates the survival of pancreatic β-cells in the process of metabolic stress (12). However, the underlying mechanism of JunD affecting pancreatic β-cells function is still unclear.

In addition, it is reported that JunD also regulates triglyceride (TG) metabolism. In metabolic cardiomyopathy models, JunD binds to peroxisome proliferators-activated receptor (PPAR) γ promoter directly, thus enabling the transcription of genes involved in the process of TG synthesis, uptake, hydrolysis, and storage (13). Besides, JunD has been proved to affect hepatic TG metabolism and non-alcoholic fatty liver disease (NAFLD) (14). Here, we investigated the role of JunD in the process of lipid accumulation and insulin secretion in pancreatic β-cells.

## Methods

### Animals

Twenty eight-week-old male C57BL/6J mice were purchased from the Model Animal Research Center of Shandong University, Jinan, China. The mice were housed in a temperature- and the humidity-controlled environment under a 12h light:12h dark cycle. After one week of adaptive feeding, the mice were given a 60% high-fat diet (HFD) for 16 weeks, whereas the control group was fed with a normal chow diet. T2DM mice model was induced by HFD combined with STZ. The HFD mice were injected intraperitoneally with streptozocin (100mg/kg, S0130; Sigma-Aldrich) dissolved in a 50 mM citric acid buffer after fasting for 12 h, while the control mice were injected with the citric acid buffer. T2DM mice were identified as two consecutive fasting glucose ≥ 16.7 mmol/L.

### Animal Procedures

Fasting blood glucose and body weight was measured once a week. The intraperitoneal glucose tolerance test (IPGTT; 2 g/kg glucose) and intraperitoneal insulin tolerance test (IPITT; 0.75 U/kg insulin) were performed 1 week after the establishment of the T2DM mice model. After glucose or insulin injection, blood glucose concentrations were measured at 0, 30, 60, 90, 120, and 180 min. The body fat mass of the mice was detected by dual-energy X-ray absorptiometry before the mice were anesthetized for euthanasia. 6 weeks after the establishment of the T2DM mice model, the mice were euthanized. Some pancreases were digested to extract islets. Other pancreases were fixed in 4% paraformaldehyde and make paraffin sections. The sections were used for TUNEL staining and immunofluorescence staining to detect the expression of insulin and glucagon.

All experimental procedures performed in this study followed the ethical guidelines for animal studies and were approved by the Institutional Animal Care and Qilu Hospital of Shandong University, China.

### Cell Culture and Treatments

INS-1 cell line was obtained from Nanjing Medical University, PR China. Cells were cultured in RPMI-1640 medium (Gibco) supplemented with 15% FBS, 10mM HEPES (Sigma-Aldrich, St. Louis, MO), 1mM sodium pyruvate (Sigma-Aldrich), 2mM L-glutamine (Gibco) and 50 μmol/L β-mercaptoethanol (Sigma-Aldrich) at 37°C with 5% CO2. Cells were cultured in a medium containing 0.4 mmol/L palmitic acid (PA, Sigma-Aldrich, USA) for 24h to induce lipotoxicity. After PA stimulation for 24h, the TUNEL staining, Oil Red staining, Western blots, and RNA extraction were performed.

### PA Preparation

0.08g sodium hydroxide (NaOH) was dissolved in 4ml ddH2O to prepare 500mmol/L NaOH solution. Then, 0.1923g PA was added into 1.5 mL 500mmol/L NaOH solution and dissolved in a water bath at 75°C to prepare a 500mmol/L PA solution. Next, 1g BSA without fatty acid was added into 20mL ddH2O preheated at 55°C and centrifuged at 8000rpm for 20min to prepare 5% BSA solution. Finally, 1ml PA solution was dissolved in 9ml 5% BSA solution to obtain 50mmol/L PA solution.

### Cell Viability

According to the manufacturer’s instructions, INS-1 cells incubated in 96-well plates were treated with different concentration of PA (0.1, 0.2, 0.4, 0.8mM), and cell viability was assessed by Cell Counting Kit-8 (CCK-8, DoJinDo, Japan) at 6, 12, 24, 36, and 48 hours. The absorbance was detected by a microplate reader at a test wavelength of 450 nm.

### Small Interfering RNA (siRNA) Transfections

The sequences of small-interfering RNAs (siRNAs) targeting rats JunD were designed and synthesized by GenePharma (Shanghai, China) for RNA silencing. The sense and antisense sequences of JunD siRNA were 5′‐GCAGUUCCUCUACCCUAAGTT‐3′. The normal control siRNA targeted the following sequence: 5′‐CUCUGAACCCUAAGGCCAATT‐3′. INS-1 cells were transfected with 160 pmol of siRNA for 6–8 h *via* Lipofectamine 2000 transfection reagent (Invitrogen, USA), according to the manufacturer’s instructions. Cells were harvested 72h later for RNA and protein.

### Glucose-Stimulated Insulin Secretion (GSIS)

After exposure to PA (0.4mM) for 24h, INS-1 cells were incubated in Krebs-Ringer bicarbonate HEPES buffer containing 2.5 mM glucose at 37°C for 1h. Then cells were treated with KRBH buffer (120 mM NaCl, 0.75 mM CaCl2·2H2O, 4mM KH2PO4,10mM NaHCO3,1mM MgSO4·7H2O, 30mM HEPES, 1% BSA) containing 25 mM glucose for an additional 1h. According to the manufacturer’s protocol, insulin concentration was measured using an insulin kit (Blue Gene, Shanghai, China). Final insulin content was normalized to the protein concentration of cells.

### Islet Extraction

After euthanasia, pancreases were isolated. First, each pancreas was added to 1× Hank’s Balanced Salt Solution (HBSS) (CC014; Macgene) containing 1.5 mg/mL collagenase V (C8170; Solarbio) and 62.5 U/mL DNase I (EN0521; Thermo Fisher Scientific). Next, the solution was shaken at 37°C with a constant temperature shaker. The digestion was terminated with pre-cooled HBSS containing 1% FBS when the tissue was visually observed as a fine line, and islets were purified through programmed sedimentation. Finally, isolated islets were handpicked and cultured in RIPM 1640) in 95% air/5% CO2 at 37°C.

### Western Blot

INS-1 cells and islets were harvested and lysed in RIPA buffer (Beyotime, China). Protein concentrations were detected with a BCA assay kit (P0012S, Beyotime, Shanghai, China). 20 micrograms of protein were loaded onto the gel. After running on 10% sodium dodecyl sulfate-polyacrylamide gel electrophoresis gels (EpiZyme, China), proteins were transferred to polyvinylidene difluoride membranes (Millipore, Temecula, CA), which were blocked with 5% milk at room temperature for 1 h. Transferred membranes were incubated overnight at 4°C with the following primary antibodies. After incubation with horseradish-peroxidase-labeled secondary antibodies, protein bands were exported by Image Lab software (BioRad, USA). Protein-band intensities were measured *via* ImageJ and were normalized to β-actin. Primary antibodies are listed in **Table 1**.

**Table 1:** Antibodies used in this study.

Antibody	Manufacture	Dilution ratio	Origin	Use	Catalog no
β-actin	CST	1:1000	USA	WB	4970
JunD	Abcam	1:1000	USA	WB	ab181615
PPARγ	Novus	1:1000	USA	WB	NBP2-76958
SREBP1c	Proteintech	1:1000	China	WB	14088-1-AP
cleaved-caspase3	CST	1:1000	USA	WB	9661
caspase3	CST	1:1000	USA	WB	9662
Bax	CST	1:1000	USA	WB	2772S
Insulin	Proteintech	1:1000	China	IF	15848-1-AP
Glucagon	Proteintech	1:200	China	IF	15954-1-AP

PPARγ, Peroxisome proliferators-activated receptor γ ; SREBP1c, Sterol regulatory element-binding protein 1c; Bax, BCL2-associated X.

### RNA Extraction and Quantitative Real-Time PCR

Total RNA from INS-1 cells was extracted with RNAiso Plus solution (Takara, Japan). Then, 1 ug RNA was reverse-transcribed into cDNA using PrimeScriptTM Reverse Transcriptase (Takara, Japan). Real-time PCR was conducted with the SYBR Green PCR kit (Takara, Japan). Relative expression levels of target mRNAs were normalized to β-actin and were calculated based on the 2-ΔΔCt comparative method. Primer sequences are listed in **Table 2**.

**Table 2:** Sequences used in this study.

Primers	Sense sequence (5’-3’)	Antisense sequence (5’-3’)	Species	Gene ID
β-actin	AGCCATGTACGTAGCCATCCA	TCTCCGGAGTCCATCACAATG	Mouse	11461
JunD	GTGCCCAGGAACTCAGAGAG	TAAAGGAAAGGCAGGGTTTG	Mouse	16478
PPARγ	TCGCTGATGCACTGCCTATG	GAGAGGTCCACAGAGCTGATT	Mouse	19016
Scd1	AGATCTCCAGTTCTTACACGACCAC	GACGGATGTCTTCTTCCAGGTG	Mouse	20249
Fas	ACCTCCAGTCGTGAAACCAT	CTCAGCTGTGTCTTGGATGC	Mouse	14104
Plin5	TGTCCAGTGCTTACAACTCGG	CAGGGCACAGGTAGTCACAC	Mouse	66968
Cd36	ATGGGCTGTGATCGGAACTG	GTCTTCCCAATAAGCATGTCTCC	Mouse	12491
Lpl	GCGAGAACATTCCCTTCACC	AGTCTCTCCGGCTTTCACTC	Mouse	16956
Fabp4	AAGGTGAAGAGCATCATAACCCT	TCACGCCTTTCATAACACATTCC	Mouse	11770
Pdx1	AACCGTCGCATGAAGTGGAA	CGAGGTTACGGCACAATCCT	Mouse	18609
Nkx6.1	GGGCTCGTTTGGCCTATTCGTT	CCACTTGGTCCGGCGGTTCT	Mouse	18096
Irs-2	CTACCCACTGAGCCCAAGAG	CCAGGGATGAAGCAGGACTA	Mouse	384783
Glut2	TCAGAAGACAAGATCACCGGA	GCTGGTGTGACTGTAAGTGGG	Mouse	20526
Ucp2	TCCTGAAAGCCAACCTCATGA	CAATGACGGTGGTGCAGAAG	Mouse	22228

PPARγ, Peroxisome proliferators-activated receptor γ ; Scd1, stearoyl-CoA desaturase 1; Fas, Fatty acid synthase; Plin5, Perilipin 5; Lpl, lipoprotein lipase; Fabp4, Fatty acid-binding protein 4; Pdx1, Pancreatic and duodenal homeobox 1; Nkx6.1, NK homeobox gene 6.1; Irs-2, Insulin receptor substrate-2; Glut2, Glucose transporter 2; Ucp2, Uncoupling protein 2.

### Oil Red O Staining

INS-1 cells were processed by Oil red O (Sigma-Aldrich, USA) staining to assess lipid content. First, cells were fixed with 4% paraformaldehyde for 15 min. Second, after two washes in PBS, cells were stained with Oil red O for 30 min at room temperature. Last, cells were treated with 60% isopropanol to differentiate the background and dyed with hematoxylin for 1 min before microscopic examination. Quantification of relative lipid content was performed by ImageJ. We counted stained lipid droplets in 100 cells.

### Immunofluorescence

Pancreatic sections were incubated with 5% BSA for 1h at room temperature and then incubated overnight with the anti-insulin (Proteintech, 15848-1-AP), and anti-Glucagon (Proteintech, 15954-1-AP) at 4°C. The next day, sections were stained with the secondary antibody for 1 hour at room temperature in the dark. The nucleus was stained with 4, 6 diamidino-2-phenylindole (DAPI) at room temperature for 5 minutes. The tissue sections and cells were imaged under a fluorescence microscope (BX61, Olympus, Japan).

### Terminal Deoxynucleotidyl Transferase-Mediated dUTP-Biotin Nick End Labeling (TUNEL) Assay

TUNEL assays of INS-1 cells were performed using the TUNEL Apoptosis Detection Kit (KGA702, KeyGEN BioTECH, China) and detected according to the manufacturer’s instructions. Briefly, INS-1 cells were fixed with 4% paraformaldehyde for 20 minutes. A 50 μL reaction mixture containing 45 μL Equilibration Buffer, 4 μL TdT Enzyme, and 1μL Biotin-11-dUTP was then added to each sample for 60 min incubation at 37°C. Then the sections and cells were washed with PBS three times and incubated with Streptavidin-TRITC for 30 min at 37°C, and finally counterstained with DAPI for 5 min. The tissue sections and cells were imaged under a fluorescence microscope (BX61, Olympus, Japan).

### Statistical Analysis

Three independent experiments were performed, and results were expressed as the mean ± the standard error of the mean (SEM). Data were compared using paired Student t-tests or one-way ANOVA followed by Bonferroni tests in GraphPad Prism 8 software (San Diego, CA, USA). P-values determined from different comparisons < 0.05 were considered statistically significant and are indicated as follows: *P < 0.05; **P < 0.01; ***P < 0.001.

## Results

### JunD Was Activated in Islets of T2DM Mice and PA-Stimulated INS-1 Cells

**Figure 1:**
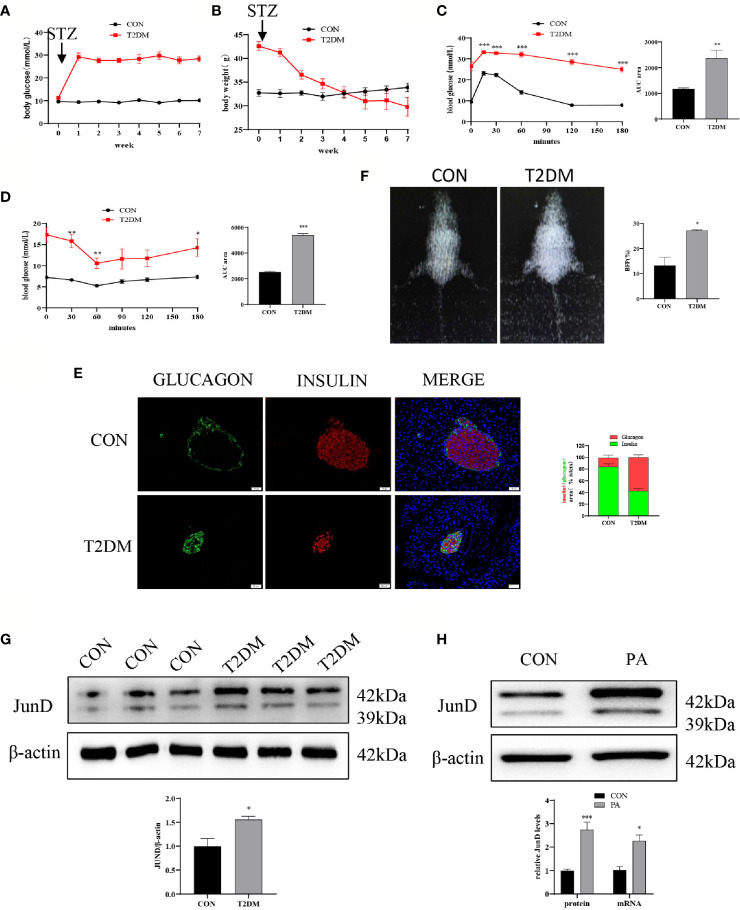
JunD was activated in islets of T2DM mice and PA-stimulated INS-1 cells. Blood glucose **(A)** and body weights **(B)** of T2DM mice compared with control mice before or after STZ injection. Intraperitoneal glucose tolerance test (IPGTT) **(C)** and intraperitoneal insulin tolerance test (IPITT) **(D)** were performed 1 week after the establishment of the T2DM mice model, and the area under curve (AUC) was also calculated. **(E)** The representative dual-energy X-ray absorptiometry image showed the body fat of the mice and the comparison of body fat percent (BFP%). **(F)** The representative immunofluorescence images of pancreases stained with insulin and glucagon, and the percentage immune-positive area of the islet insulin and glucagon, scale bar=20 μm. **(G)** The protein expression of JunD in islets was detected by Western blot. **(H)** The protein mRNA levels of JunD in PA-stimulated INS-1 cells. Data are expressed as the mean ± SEM. *p < 0.05; **p <0.01; ***p < 0.001 (compared with control group).

First, we evaluated the establishment of the T2DM mice model. The body weight and fasting blood glucose were measured once a week. As shown in **Figure 1A**, the blood glucose of T2DM mice was higher than that of control mice. Meanwhile, the body weights of mice in two groups were measured. The results showed that the body weights of T2DM mice were significantly lower than that of blank controls (**Figure 1B**). Glucose homeostasis of T2DM mice was significantly impaired. The IPGTT showed significant and glucose intolerance (**Figure 1C**), and the IPITT indicated markedly reduced insulin sensitivity (**Figure 1D**) in T2DM mice. The immunofluorescence showed that T2DM mice had a lower insulin-positive cell ratio and a higher glucagon-positive cell ratio (**Figure 1E**). We performed body composition analysis by dual-energy X-ray absorptiometry to study the body fat percentage (BFP, %) levels. The results showed that the BFP levels of T2DM mice were dramatically elevated compared with the control group (**Figure 1F**). These data suggested that the T2DM mice model was successfully established. In addition, the protein level of JunD in islets of T2DM mice was elevated compared to that of control mice (**Figure 1G**), which indicated the activation of JunD.

Then we evaluated whether JunD was activated in PA-induced INS-1 cells. PA is widely used to induce lipotoxicity mimicking the environment of T2DM (17). The CCK8 assay was performed to determine the concentration and stimulation time of PA (**Figure S1A**). We examined whether 0.4mM PA could activate JunD. We found that the expression of JunD was increased at 6h, and reached its peak at 24h, then gradually decreased (**Figure S1B**). As a result, we chose a 0.4 mM PA to stimulate for 24h in the following experiment. The results showed a significant increase in the expressions of JunD in PA-treated INS-1 cells compared with control cells, both at mRNA and protein levels (**Figure 1H**). Taken together, these results confirmed the activation of the JunD in islets of T2DM mice and PA-treated INS-1 cells.

### PA Induced INS-1 Cells Dysfunction

**Figure 2:**
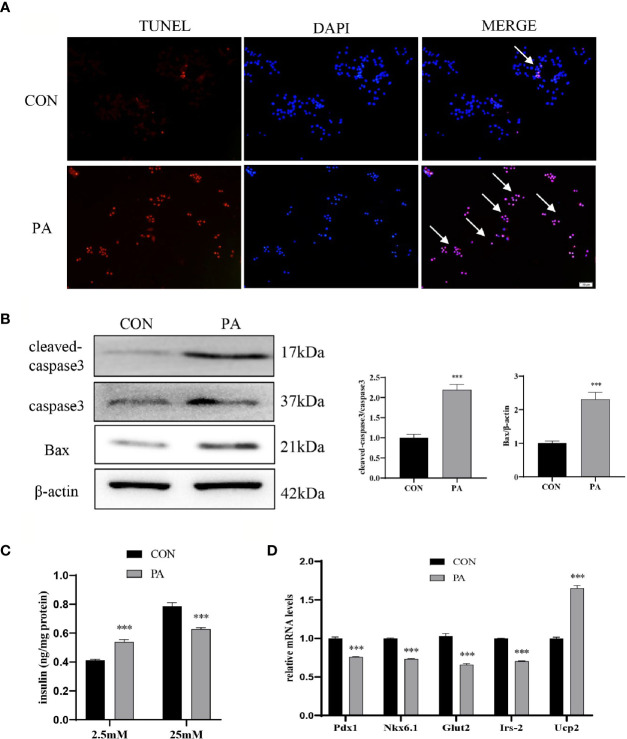
PA induced INS-1 cells dysfunction. INS-1 cells were incubated with PA (0.4mM) for 24h. **(A)** Apoptosis was assessed by terminal deoxynucleotidyl transferase-mediated dUTP-biotin nick end labeling (TUNEL) assay, scale bar=50 μm. **(B)** The protein expressions of cleaved-caspase3 and Bax were detected by Western blot. **(C)** Glucose-stimulated insulin secretion (GSIS) was performed to show the dysfunction of insulin secretion after PA stimulation. **(D)** The mRNA expressions of insulin secretion-related genes, including Pdx1, Nkx6.1, Irs-2, Glut2, and Ucp2. Data are expressed as the mean ± SEM. ***p < 0.001 (compared with control group).

The TUNEL assay showed that the number of TUNEL-positive INS-1 cells after PA stimulation was dramatically increased compared with blank controls (**Figure 2A**). Additionally, we examined the protein levels of cleaved-caspase3 and Bax. The results showed that the expressions of cleaved-caspase3 and Bax were increased in PA-induced INS-1 cells, which indicated that the apoptosis of INS-1 cells was increased under PA stimulation (**Figure 2B**). Insulin secretion of INS-1 cells was measured by glucose-stimulated insulin secretion (GSIS) after exposure to PA for 24h. The results showed that PA upregulated basal insulin secretion at 2.5mM glucose. However, under the circumstance of 25mM glucose, insulin secretion after PA stimulation was much lower than that of the control group (**Figure 2C**). Meanwhile, essential genes for pancreatic β-cells function, such as Pdx1, Nkx6.1, Irs-2, Glut2, and Ucp2 were evaluated. Compared with the control group, the mRNA expressions of Pdx1, Nkx6.1, Irs-2, and Glut2 were significantly reduced after PA stimulation. On the other hand, PA increased Ucp2 mRNA levels in INS-1 cells (**Figure 2D**), which inhibits insulin secretion by reducing ATP synthesis (16). Collectively, these findings indicated that lipotoxicity led to the dysfunction of pancreatic β-cells.

### PA Induced Lipid Accumulation in INS-1 Cells

**Figure 3:**
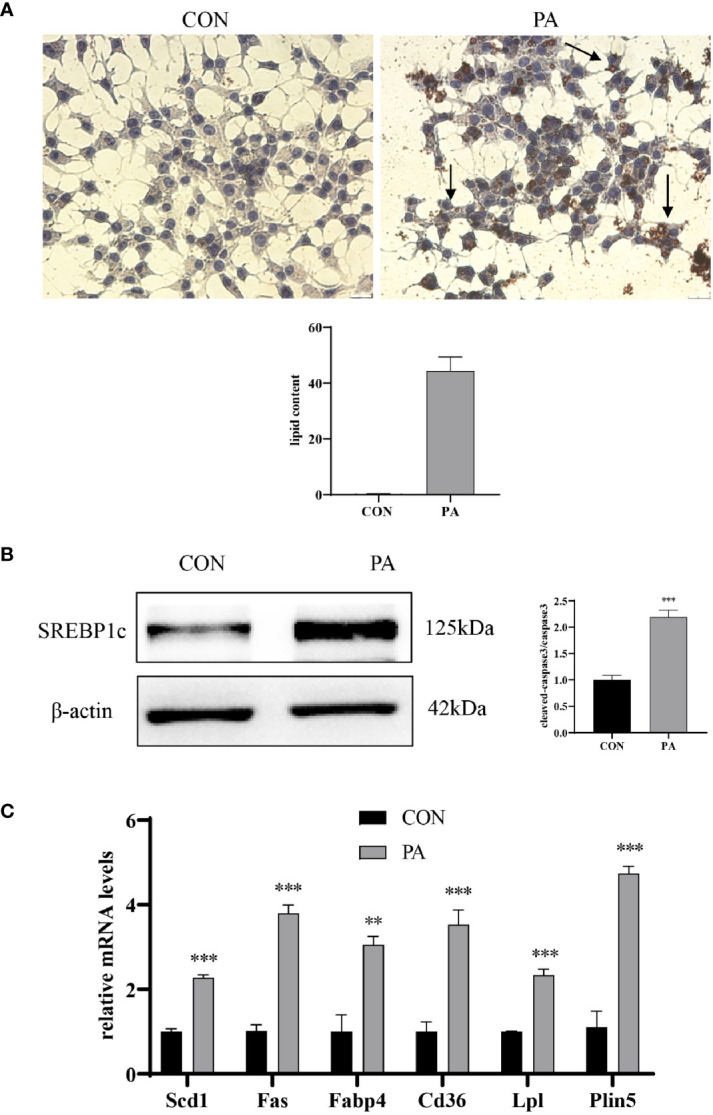
PA induced lipid accumulation in INS-1 cells. **(A)** Oil red O staining was performed to detect the intracellular lipid accumulation, scale bar=20 μm. Quantification of lipid content was performed by ImageJ. **(B)** The protein expression of SREBP1c was detected by Western blot. **(C)** The mRNA levels of TG–synthesis, uptake, hydrolysis, and storage-related genes, including Fas, Scd1, Cd36, Fabp4, Lpl, and Plin5. Data are expressed as the mean ± SEM. **p < 0.01; ***p < 0.001 (compared with control group).

We further assessed whether PA could induce lipid accumulation in INS-1 cells. Intracellular lipid accumulation was evaluated by Oil red O staining. As shown in **Figure 3A**, there were no apparent lipid droplets in the control group. In contrast, after exposure to PA, a large number of lipid droplets were accumulated in the cytoplasm. SREBP1c, a transcription factor responsible for fatty acid synthesis (17), was increased in PA-stimulated INS-1 cells (**Figure 3B**). The real-time PCR array revealed a profound upregulation of genes implicated in fatty acid synthesis (i.e., Fas, SCD1), uptake (i.e., Cd36, Fabp4), hydrolysis (i.e., Lpl), and storage (i.e., Plin5) after PA stimulation compared to the control group (**Figure 3C**). These results indicated that PA stimulated lipid production and led to lipid accumulation in INS-1 cells.

### JunD/PPARγ Signaling Pathway Involved in PA-Induced INS-1 Cells Dysfunction

**Figure 4:**
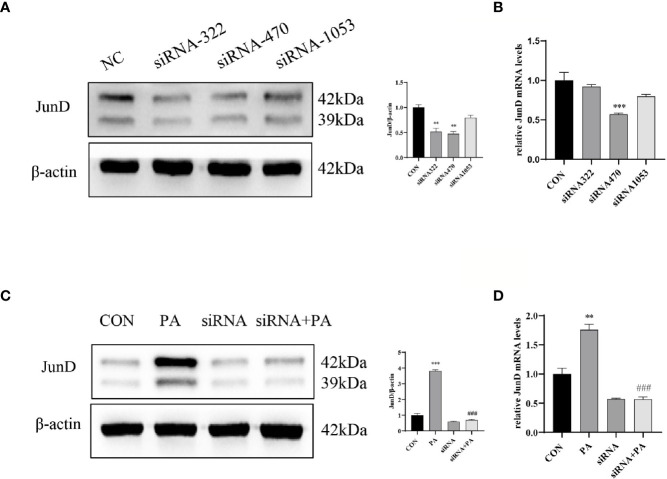
The inhibition efficiency of JunD. **(A)** Western blot analyses and real-time PCR **(B)** for inhibition efficiency of JunD in INS-1 cells. **(C)** The protein and **(D)** mRNA levels of JunD. Data are expressed as the mean± SEM. **p < 0.01; ***p < 0.001 (compared with control group), ^###^p < 0.001 (compared with PA group).

**Figure 5:**
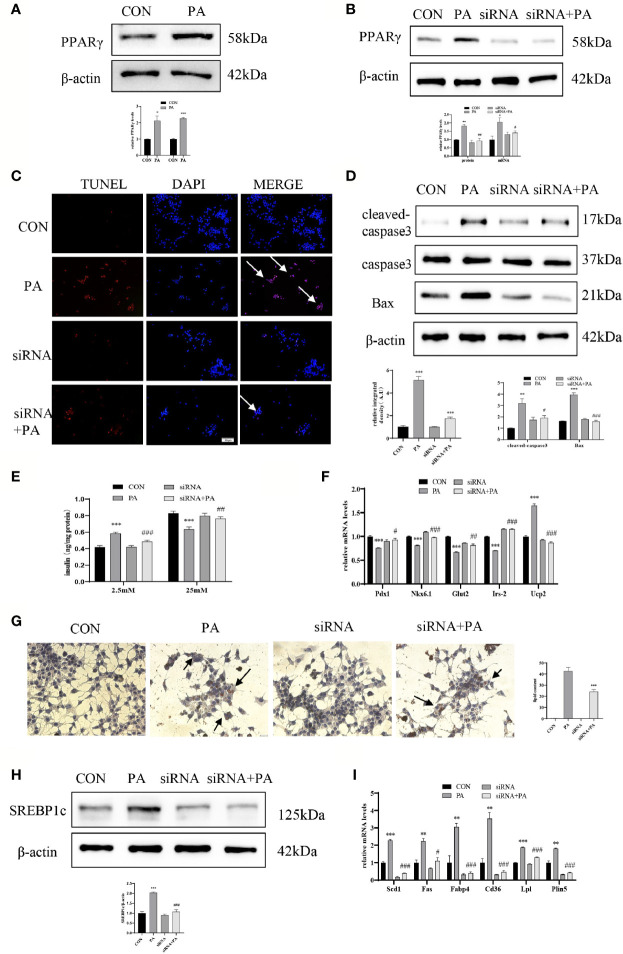
JunD/PPARγ signaling pathway involved in PA-induced INS-1 cells dysfunction. INS-1 cells were transfected with JunD siRNA 470 followed by treatment with 0.4 mmol/L PA for 24 hours. **(A, B)** Expressions of PPARγ in protein and mRNA levels. **(C)** Apoptosis was assessed by TUNEL assay, scale bar=50 μm. **(D)** The protein expressions of cleaved-caspase3 and Bax were detected by Western blot. **(E)** GSIS was performed to show the dysfunction of insulin secretion after PA stimulation. **(F)** The mRNA expressions of insulin secretion-related genes, including Pdx1, Nkx6.1, Irs-2, Glut2, and Ucp2. **(G)** Oil Red O staining was performed to detect the intracellular lipid accumulation, scale bar=20 μm. **(H)** The protein expression of SREBP1c was detected by Western blot. **(I)** The mRNA levels of TG synthesis, uptake, hydrolysis, and storage-related genes, including Fas, Scd1, Cd36, Fabp4, Lpl, and Plin5. Data are expressed as the mean± SEM. *p < 0.05; **p < 0.01; ***p < 0.001 (compared with control group), ^#^p < 0.05, ^##^p < 0.01, ^###^p < 0.001 (compared with PA group).

To investigate the effect of JunD on the PA-induced INS-1 cells dysfunction, the JunD knockdown model in INS-1 cells was established. Gene silencing of JunD by siRNA was confirmed by Western blot and real-time PCR (**Figures 4A–D**). Our results showed that PPARγ was increased after PA stimulation, both at protein and mRNA levels (**Figure 5A**). JunD depletion downregulated the expressions of PPARγ (**Figure 5B**), which indicated that PPARγ might be the downstream target of JunD.

Then we evaluated the function and lipid accumulation of INS-1 cells. The TUNEL assay showed that the number of TUNEL-positive cells was decreased (**Figure 5C**), and Western blot showed lower expressions of cleaved-caspase3 and Bax (**Figure 5D**) in PA-induced INS-1 cells after transfection with JunD siRNA. As shown in **Figure 5E**, gene silencing of JunD reversed the impaired GSIS after PA stimulation. Meanwhile, the mRNA levels of Pdx1, Nkx6.1, Irs-2, Glut2, and Ucp2 were also significantly ameliorated (**Figure 5F**). The Oil Red O staining showed reduced lipid droplets in JunD-depleted INS-1 cells (**Figure 5G**). Depletion of JunD also suppressed the expression of SREBP1c (**Figure 5H**), as well as the levels of Scd1, Plin5, Lpl, Fas, Cd36, and Fabp4 (**Figure 5I**). These results revealed that JunD/PPARγ signaling pathway was involved in the dysfunction of INS-1 cells.

## Discussion

Pancreas plays a major role in maintaining normal blood glucose levels; however, the islet function of T2DM patients is impaired. Previous studies have shown that metabolic stress leads to the disorder of glucose and lipid metabolisms and finally results in cell dysfunction (18). The dysfunction of pancreatic β-cells is mainly manifested by insufficient secretion, which leads to accelerated progress of diabetes and forms a vicious circle (19); and if without timely intervention, the body will gradually lose weight (20, 21), leading to many serious complications. The weight loss is likely to be the result of catabolic effects of insulin deficiency and acidosis (21, 22); meanwhile, if T2DM progresses to diabetic nephropathy, osmotic diuresis can also lead to blood volume reduction and weight loss (23). Therefore, restoring the function of pancreatic β-cells is a key node to treat T2DM. High-fat diet combined with STZ was used to mimics T2DM model. High-fat diet is used to simulate the eating habits of most patients with type 2 diabetes (24), and STZ helps to destroy the function of β cells (25). The expansion of adipose tissue releases a large amounts of nonesterified fatty acids, such as oleic acid and PA (26). PA induces insulin resistance and pancreatic β-cells dysfunction *via* three mechanisms (27): (1) Increased internalization of palmitic acid results in lipotoxicity (28); (2) The excess of PA results in the endoplasmic reticulum and mitochondria dysfunction (29, 30); (3) PA can activate toll-like receptor (TLR)-4 and high-fat diets activate the IKKβ–NF-κB pathway, leading to an inflammatory environment (31, 32). Here, we revealed the important role of JunD in pancreatic β-cells by altering lipid accumulation.

The causes of pancreatic β-cells dysfunction include ectopic lipid accumulation, which leads to oxidative stress, inflammation, and β-cell apoptosis (33). Even more notably, improving β-cell lipid metabolism could boost the regeneration of β-cells (34). Therefore, it is important to decipher the mechanism of lipid deposition in β cells. Most of the studies focus on the disorder of lipid synthesis, transport, hydrolysis, and storage. Estrogen receptors and liver X receptors are both expressed in pancreatic β-cells and regulate genes related to lipid metabolisms, such as Fas, Acc, and Cpt1a (35, 36). Autophagy is a lysosomal-dependent cellular catabolism mechanism and decreases cellular lipid stores in pancreatic β-cells (37). Adipose triglyceride lipase is responsible for lipid droplet mobilization in human β-cells (38). However, the researches on the lipid metabolism of β-cells are still insufficient and there are few targeted treatments.

The AP-1 transcription factor JunD regulates various target genes involved in cell growth, proliferation, and apoptosis (9, 39). Recently, JunD has emerged as a vital player in the setting of metabolic diseases (11–14, 40). Hyperglycemia promotes ROS production by downregulating JunD expression, which leads to cardiac dysfunction. In addition, previous studies have indicated that the impact of JunD in metabolic cardiomyopathy and NAFLD is mainly caused by promoting intracellular lipid deposition through the PPARγ signaling pathway. JunD−/− mice showed reduced intra-myocardial TG accumulation, total liver, and fat pad weights (13, 14). Besides, the cardiac specimens of obese patients had higher expression of JunD, as well as TG-related genes *vs*. non-obese hearts (13). These researches indicated that JunD is an upstream regulator of PPARγ and mediates the transcription of genes involved in lipid uptake, hydrolysis, and storage.

JunD also acts as a stress-responsive factor that induces redox imbalance and apoptosis in pancreatic β-cells (12). However, it will be of interest to determine whether JunD is involved in the lipid accumulation of pancreatic β-cells. Our study suggested that JunD was activated in PA-stimulated INS-1 cells, and JunD depletion prevented PA-induced impaired GSIS, lipid accumulation. The research on the function of PPARγ on pancreatic β-cells is contradictory. Many studies have shown that PPARγ activation induces insulin secretion through proliferation (41), anti-apoptosis (42), or antioxidation (43). Hong et al. indicated that PPARγ agonist could attenuate PA-induced inflammation and ER stress in pancreatic β-cells (44). However, our results showed that PPARγ was activated under PA stimulation and modulate the upregulation of genes involved in lipid metabolism, which was consistent with the studies performed by Hogh et al. Hogh et al. found that overexpression of PPARγ specifically in pancreatic β-cells alters islet lipid metabolism and exacerbates β-cells dysfunction (45). Ectopic expression of PPARγ in INS-1 cells increases lipid accumulation and decreases GSIS (46). Peroxisome-generated hydrogen peroxide mediates the lipotoxicity in pancreatic β-cells, which might be the potential mechanism of β cell dysfunction caused by PPARγ activation (47). Under pathological states, such as high glucose and obesity, the activation of PPARγ in pancreatic β-cells might aggravates apoptosis and affect glucose homeostasis (48).

It is unclear whether other mechanisms are contributing to the regulation of JunD in GSIS. Mitochondrial dysfunction (49), ER stress (50), as well as imbalance of Ca2+ homeostasis (51) are associated with pancreatic β-cells dysfunction. Mitochondrial dysfunction including changed mitochondrial structure, decreased mitochondrial respiration, reduced mitochondrial ATP production (52). Akhmedov et al. indicated that cardiomyocyte-specific JunD overexpression reduced Sirt3 transcription, thus leading to mitochondrial dysfunction (53). Our results showed that JunD reversed the increase of Ucp2 induced by PA, which inhibits insulin secretion by reducing ATP synthesis (16), indicating that JunD might improve GSIS by changing mitochondrial ATP production. Good et al. found that the depletion of JunD downregulated Ptgs2 in db/db mice, which encodes cyclooxygenase-2 (COX2) and imparts insulin secretion of pancreatic β-cells (12).

Other AP-1 components also play an important role in regulating pancreatic β-cells. In β cells, glucose modulates the expression pattern of fos and jun genes, which could induce an immediate-early gene c-fos (54, 55). The immediate-early genes encode transcription factors and regulate downstream target genes (56). Human amylin could activate the expression of c-jun, mediate the combination of c-jun with c-fos or ATF-2, and activate the downstream apoptosis pathway of pancreatic β-cells (57). Gurzov et al. indicated that JunB plays a protective role against apoptosis in pancreatic β-cells through inhibiting iNOS and Chop expression (58). Meanwhile, an inflammatory environment upregulates JunB/ATF3 pathway and protects β cells by increasing cAMP expression (59).

Taken together, our research provides a new strategy for restoring the function of pancreatic β-cells and has a prospect of clinical treatment.

## Data Availability Statement

The original contributions presented in the study are included in the article/**Supplementary Material**. Further inquiries can be directed to the corresponding authors.

## Ethics Statement

The animal study was reviewed and approved by Shandong University.

## Author Contributions

KXW, YXC, and ZNY were involved in study design, interpreting data, statistical analysis, creating tables and figures, and writing the manuscript. KXW, PL, and YS were involved in interpreting data, statistical analysis, and designed the research, supervised the work. All authors contributed to the article and approved the submitted version.

## Funding

This work was supported by the National Natural Science Foundation of China (82070852, 81873650, 82070799), the Natural Science Foundation of Shandong Province (ZR2020MH105) and the fundamental research funds of Shandong University (2018JC015).

## Conflict of Interest

The authors declare that the research was conducted in the absence of any commercial or financial relationships that could be construed as a potential conflict of interest.
