# Supplementary material for: JunD Regulates Pancreatic β-Cells Function by Altering Lipid Accumulation
Source: Front Endocrinol (Lausanne). 2021 Jul 16;12:689845. doi: 10.3389/fendo.2021.689845 (PMC8322846; doi:10.3389/fendo.2021.689845)
Supplement: Supplementary file 1 [file DataSheet_1.docx]

**
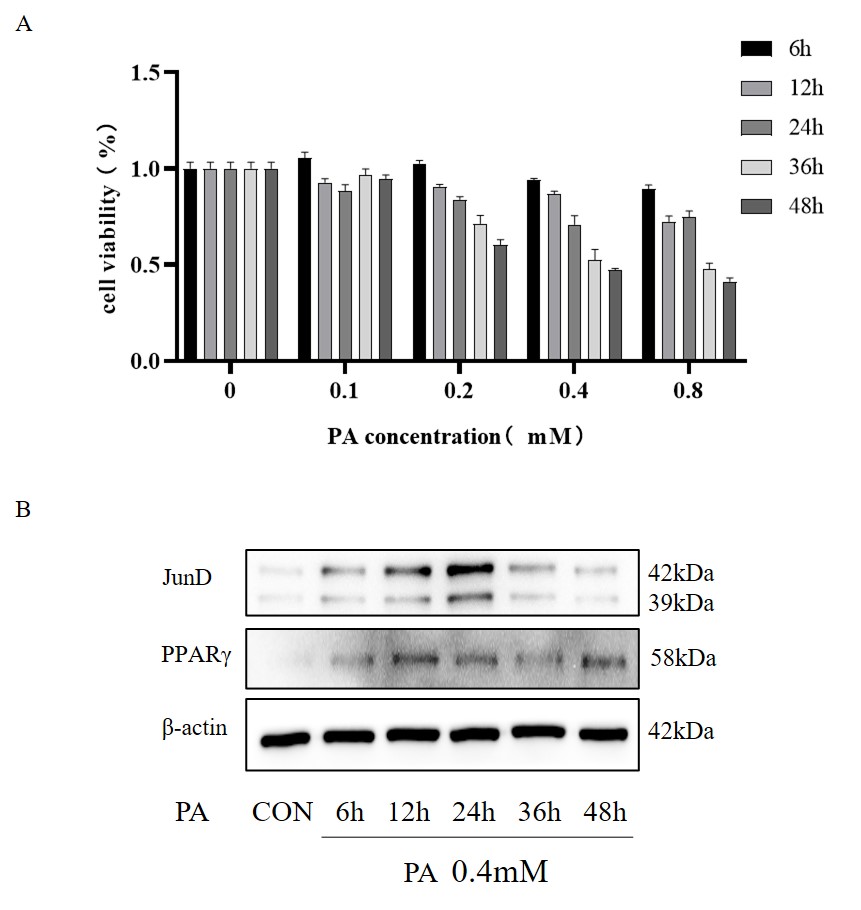
****Supplementary Figure 1.** The cell counting kit 8 (CCK8) assay. (S1A) The concentration (0.1, 0.2, 0.4, 0.8mmol/L) and stimulation time (6, 12, 24, 36, 48h) of palmic acid (PA) to induce lipotoxicity in INS-1 cells. (B) the expression of JunD and PPARγ when it was incubated with PA (0.4mM).


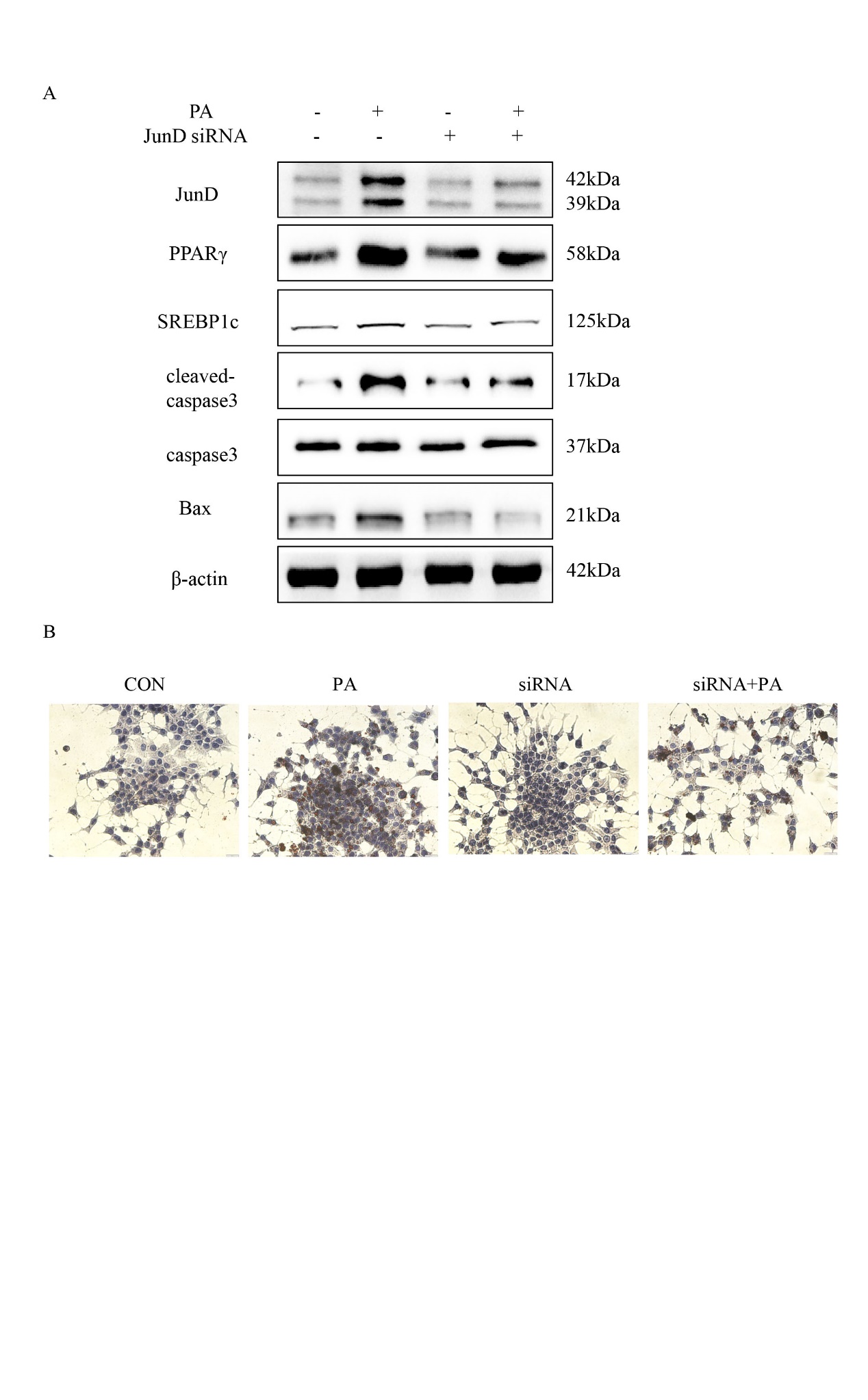


**Supplementary Figure 2.** INS-1 cells were transfected with JunD siRNA 322 followed by treatment with 0.4 mmol/L PA for 24 hours. (A) The expression of JunD, PPARγ, SREBP1c, cleaved-caspase3, caspase3, Bax was evaluated after transfected with siRNA322. (B) Oil Red O staining was performed to detect the intracellular lipid accumulation.
